# Pyogenic Sacroiliitis in Children: Two Case Reports

**DOI:** 10.1155/2012/415323

**Published:** 2012-07-05

**Authors:** L. Ghedira Besbes, S. Haddad, A. Abid, Ch. Ben Meriem, M. N. Gueddiche

**Affiliations:** ^1^Pediatric Department, Fattouma Bourguiba Hospital, 5000 Monastir, Tunisia; ^2^Service de Pédiatrie, CHU, 5000 Monastir, Tunisia; ^3^Orthopedic Department, Fattouma Bourguiba Hospital, Monastir, Tunisia

## Abstract

Pyogenic sacroiliitis is rare and accounts for approximately 1-2% of osteoarticular infections in children. Considerable delay between presentation and diagnosis is recognized. Two cases of pyogenic sacroiliitis are described. The first case is a 28-month-old girl presented with acute onset of fever, pain in the left hip, and limpness. Computed tomography (CT), bone scans, and magnetic resonance imaging (MRI) of the pelvis showed characteristic findings of infectious sacroiliitis, and blood cultures were negatives. The second case is a 13-year-old girl presented with acute onset of fever, pain in the right hip, and buttock, with inability to walk. The diagnosis of pyogenic sacroiliitis was confirmed by bone scans, and CT of the pelvis and blood cultures have identified *Proteus mirabilis*. The two children recovered fully after 6 weeks of antimicrobial therapy. Pyogenic sacroiliitis is an uncommon disease in children. The key to successful management is early diagnosis in which CT, bone scans, and MRI findings play a crucial role. If the diagnosis is established promptly, most patients can be managed successfully with antimicrobial therapy.

## 1. Introduction 

 Pyogenic sacroiliitis is relatively rare, representing only 1-2% of all cases of septic arthritis in children [1]. Initial symptoms are usually nonspecific and difficult to differentiate from septic arthritis of the hip. Diagnosis of pyogenic sacroiliitis has been difficult in the past due to its deep location and may be delayed due to the lack of specific clinical signs. Delay in diagnosis may lead to several complications, such as abscess or sequestration formation, prolonged period of sepsis, and long-term joint deformity [2]. Newer diagnostic techniques such as bone scanning, computed tomography (CT), and magnetic resonance imaging (MRI) aid in early diagnosis and treatment [3]. We present two cases of pyogenic sacroiliitis in children. 

## 2. Case Reports 

### 2.1. Patient 1

**Figure 1:**
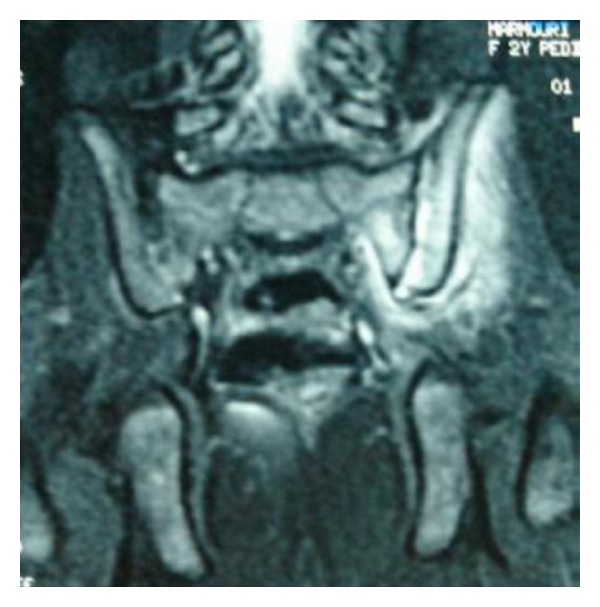
MRI of the pelvis: on T2-weighted images an increase of the signal intensities of the left sacroiliac joint and increase of the signal of the iliacus and gluteal muscles. Also there was a little effusion in the left sacroiliac joint.

A 28-month-old girl was admitted to the Pediatric Department with a five-day history of pain in the left hip, limpness, and fever. The girl had a pelvic trauma one day before the onset of the symptoms. Examination showed an irritable girl with temperature of 39°C. A general examination was normal. Although the girl kept antalgic position (semiflexed of the left leg) with pain in left groin, hyperextension of the hip, forced abduction, and external rotation of the left hip were limited and painful. Plain radiograph of the pelvis and ultrasonography of the hips were normal. Laboratory findings on admission showed a white blood cell count of 11600/*μ*L, erythrocyte sedimentation rate (ESR) of 110 mm/1st hour, and C reactive protein (CRP) of 69 mg/L. A bone scan (99 mT-MDP) performed two days after admission revealed increased uptake in the left sacroiliac joint. A CT scan performed four days after admission showed pinching of the left sacroiliac joint without effusion in this joint and thickening of the left iliacus muscles. The sacral and iliac cortices were regular along the sacroiliac joint. The left hip joint was normal without effusion. The diagnosis of pyogenic sacroiliitis was suspected, and intravenous treatment with oxacillin and gentamycin was started. MRI of the pelvis performed ten days after admission revealed on T2-weighted images an increase of the signal intensities of the left sacroiliac joint and increase of the signal of the iliacus and gluteal muscles. Also there was a little effusion in the left sacroiliac joint (Figure 1). All radiologic findings (bone scan, CT scan, and MRI) suggested a left sided sacroiliitis. Blood cultures were negative. On intravenous antibiotherapy, pain decreased, and mobility improved after three weeks. The girl was discharged after three weeks of intravenous oxacillin and continued oral oxacillin for three weeks. Followed up six months later, the girl improved well without sequelae. 

### 2.2. Patient 2

A previously healthy 13-year-old girl was admitted to the Pediatric Department presenting with fever and inability to walk. Sudden right hip and buttock pain with fever up to 40°C had developed in the patient three days before admission to the hospital. The pain had gradually progressed to the point that she was unable to walk. The patient had no history of prior trauma but she had a skin infection in her right foot secondary to tattooing. On admission to the hospital, the patient had a temperature of 39°C and marked pain on motion of the right hip with limitation of right hip movement. Physical examination revealed exquisite tenderness on palpation of the right hip and the right sacroiliac joint. 

**Figure 2:**
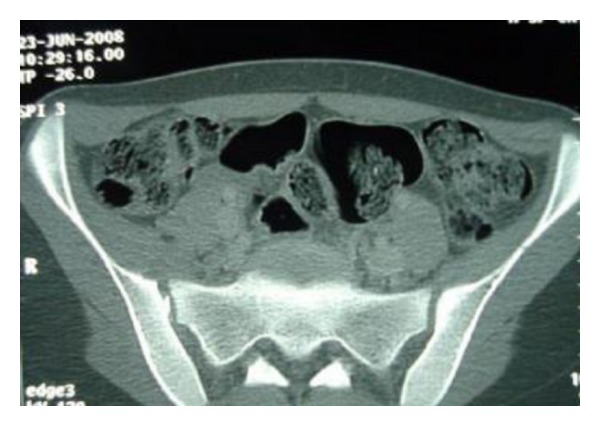
CT of the pelvis revealed apparent widening of the right sacroiliac joint with infiltration, edema of the soft tissue surrounding the joint, and presence of microabscess in the right iliacus muscle.

**Figure 3:**
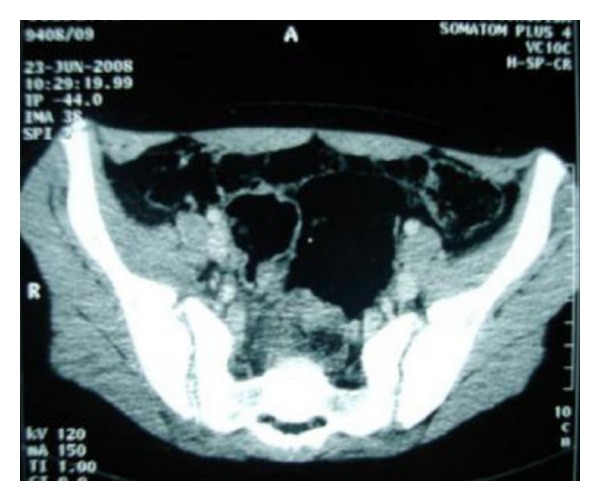
CT of the pelvis showed signs of right sacroiliitis with infiltration and microabscess of the soft tissue, surrounding the joint with erosions of the subchondral iliac bone.

Laboratory findings on admission included a white blood cell count of 31200/*μ*L with 80% polymorphonuclear leukocytes, ESR of 110 mm/1st hour, and CRP of 213 mg/L. A roentgenogram of the pelvis showed no abnormalities, and ultrasono-graphic of the hips was normal. The admitting diagnosis was septic arthritis of the right hip or right pyogenic sacroiliitis. Empiric intravenous antibiotherapy (oxacillin gentamycin) was started. On the second day of admission, CT of the pelvis was performed; it revealed apparent widening of the right sacroiliac joint with infiltration, edema of the soft tissue surrounding the joint, and presence of microabscess in the right iliacus muscle. All these findings suggested right pyogenic sacroiliitis joint (Figure 2). An isotope bone scan was performed two days after admission, showed increased uptake in the right sacroiliac joint. Three days after the onset of antibiotherapy, the patient did not respond to treatment, continued to have fever, groin pain, and since blood cultures have identified *Proteus mirabilis*, than antibiotherapy was switched to the association of Cefotaxime with fosfomycin intravenously. Over the next 48 hours, apyrexia was obtained and symptoms gradually improved. CT of the pelvis was performed fifteen days after the first CT; it showed signs of right sacroiliitis with infiltration and microabscess of the soft tissue, surrounding the joint with erosions of the subchondral iliac bone (Figure 3). The patient responded promptly to 25 days of intravenous antibiotherapy followed by an additional two weeks of oral antibiotherapy (ofloxacin). Followed up six months later the girl improved well without sequelae. 

## 3. Discussion 

Pyogenic sacroiliitis is quite rare disease in children and remains a diagnostic challenge. Schaad et al. had reported 77 cases of pyogenic sacroiliitis in patients <17 years old in a review of the literature from 1941 to 1979 [4]. Wu et al. reported a series of 33 cases of pyogenic sacroiliitis and they identified 11 cases aged less than 15 years [5]. Wada et al. reviewed eight pediatric patients with sacroiliitis identified between 2000 and 2005 [6]. Recently Molinos Quintana et al reported 11 patients aged less than 14 years who met the criteria of pyogenic sacroiliitis during eight years (2002–2010) [7]. 

Patients group at increased risk of pyogenic sacroiliitis includes children, immunosuppressed patients, and patients with sickle cell disease [8]. Trauma is an important predisposing factor with an estimated 10% of cases having a prior history of pelvic trauma [9]. Other predisposing factors include atopic dermatitis, insect bite, folliculitis and furunculosis [7]. In our first case a pelvic trauma was reported and in the second case tatooing of the foot preceded the osteoarticular infection. 

Diagnosis of osteoarticular infections in the pelvic region has been generally considered challenging due to the lack of specificity and great variety of their symptoms. The onset of disease is insidious in two-thirds of patients [7]. The typical symptoms of fever, buttock pain, and limping gait are often absent. Furthermore, because of the complex anatomy of the sacroiliac joint, the pain is commonly found to be referred to other sites such as the lower back, abdomen, thigh, or hip and can mimic a number of processes other than sacroiliitis [5]. General features of sepsis such as tachycardia and tachypnea are usually seen at initial presentation [10]. Clinical examination is decisive: sacroiliac joint palpation, FABER test (flexion, forced abduction, and external rotation) of the ipsilateral hip, and hyperextension of the hip (Gaenslen's test) are clues to diagnosis [11]. Although these provocative tests have proven to be reliable in terms of sensitivity, specificity, and predictive values in determining the source of pain in many chronic conditions, they are often not performed in the present clinical context because of a low degree of suspicion even after a careful anamnesis. 

The differential diagnosis is broad including appendicitis, tumours, discitis, and septic arthritis of the hip or clinical sepsis [10]. 

However the clinical picture may be not specific, the consequence is a considerable delay between presentation and diagnosis is recognized [7]. Most cases of pyogenic sacroiliitis are unilateral, right sacroiliac joint is more frequently involved than left side; bilateral involvement is not uncommon [5]. 

There is no specific blood test which points to the diagnosis of pyogenic sacroiliitis; white blood cells count may be increased or normal; ESR and CRP may be elevated in the majority of cases, but while they are sensitive, they may not be specific [12]. 

Blood cultures should be performed before antibiotherapy. The overall positive blood culture rate is low in pediatric patients (45.5%) [5]. Nevertheless, local synovial fluid cultures have a high yield rate for pathogens. Sacroiliac joint synovial fluid aspiration is technically difficult due to the joint being deep seated and oblique and thus relatively inaccessible [10]; this invasive procedure is not warranted routinely [5]; it is recommended in patients with clinical and radiographic features suggestive of pyogenic sacroiliitis, but with negative blood cultures, and poor responding or nonresponding to conventional antibiotic therapy [7]. The most common bacterial pathogen recovered from blood and/or from the synovial fluid specimen is *Staphylococcus aureus*, accounting for 80% of pyogenic sacroiliitis in paediatric patients. Other isolates include *Streptococcus beta-haemolyticus, Haemophilus influenzae, Escherichia coli, *and* Salmonella* [10]. *Proteus mirabilis* is rarely isolated [13]. Sacroiliitis secondary to *Streptococcus pneumoniae* is exceedingly rare; only five cases have been described to date [14]. Brucella sacroiliitis exists in endemic areas [15]. 

Plain radiographs are often initially negative. Later, widening of the joint space of the affected side, then blurring of subchondral bone, and demineralization appear [11]. 

Ultrasound has not been helpful except to exclude hip-joint effusions [16]. Isotope bone scanning is an essential tool for early diagnosis with an excellent sensitivity [3]. Unilateral increased uptake can be seen as early as 3 days after onset of symptoms [16]. 

MRI is the imaging technique with the highest sensitivity and specificity (95% and 100%, resp.) for the confirmation of the diagnosis of pyogenic sacroiliitis [17]. MRI combines good visualisation of the complicated anatomy of the sacroiliac joint with the ability to localise different degrees of inflammation and edema. It has the ability to visualise fluid in the sacroiliac joint, bone marrow edema, and soft tissue abscess [18, 19]. In sacroiliitis with local abscess formation, MRI can detect spinal involvement which is important in the decision of surgical intervention. 

Medical management of pyogenic sacroiliitis is early diagnosis, antibiotic therapy, and bed rest. Antibiotic should be direct against *Staphylococcus aureus* and intravenous oxacillin should be the drug of choice for empirical therapy followed by oral oxacillin (after normalisation of both symptoms and blood biology) for a total duration of 4–6 weeks [10]. If pathogen is identified antibiotherapy is adjusted. In cases of poor response to initial empirical antistaphylococcal therapy, the clinicians should prescribe antimicrobials with coverage of gram-negative pathogens. At present there is no clear consent regarding optimal duration of antimicrobial therapy for patients with sacroiliac joint involvement [7]. Conservative management therapy has been proved to be effective in a series of patients with soft tissue abscesses [6]. However surgical drainage is indicated in presence of sequestrum formation, osteomyelitis, and failure of medical management [10, 11]. 

Pyogenic sacroiliitis should always be included in the differential diagnosis of any child with fever and buttock, hip, or back pain. FABER test should be performed routinely in these patients and if positive, an MRI is recommended to rule out pyogenic sacroiliitis. Antibiotic therapy has proved effective in most cases with good clinical response and with no sequelae during followup.
